# Neutrophils Directly Recognize Group B Streptococci and Contribute to Interleukin-1β Production during Infection

**DOI:** 10.1371/journal.pone.0160249

**Published:** 2016-08-10

**Authors:** Nastaran Mohammadi, Angelina Midiri, Giuseppe Mancuso, Francesco Patanè, Mario Venza, Isabella Venza, Annamaria Passantino, Roberta Galbo, Giuseppe Teti, Concetta Beninati, Carmelo Biondo

**Affiliations:** 1 Department of Clinical and Experimental Medicine, University of Messina, 98125, Messina, Italy; 2 Department of Human Pathology, University of Messina, 98125, Messina, Italy; 3 Department of Veterinary Science, University of Messina, 98125, Messina, Italy; 4 Department of Biological, Chemical and Environmental Sciences, University of Messina, 98125, Messina, Italy; 5 Charybdis Vaccines Srl, 98125, Messina, Italy; 6 Scylla Biotech Srl, 98125, Messina, Italy; National University, COSTA RICA

## Abstract

Previous studies have shown that the pro-inflammatory cytokine IL-1β has a crucial role in host defenses against group B streptococcus (GBS), a frequent human pathogen, by recruiting neutrophils to infection sites. We examined here the cell types and mechanisms involved in IL-1β production during infection. Using a GBS-induced peritonitis model in mice, we first found that a large proportion of exudate cells contain intracellular IL-1β by immunofluorescence. Of the IL-1β positive cells, 82 and 7% were neutrophils and macrophages, respectively, suggesting that the former cell type might significantly contribute to IL-1β production. Accordingly, depletion of neutrophils with anti-Ly6G antibodies resulted in a significant reduction in the levels of IL-1β, but not of TNF-α or IL-6. We next found that neutrophils are capable of releasing mature IL-1β and TNF-α directly in response to *in vitro* stimulation with GBS. The production of pro-IL-1β and TNF-α in these cells required the Toll-like receptor (TLR) adaptor MyD88 and the chaperone protein UNC93B1, which is involved in mobilization of a subfamily of TLRs to the endosomes. Moreover, pro-IL-1β processing and IL-1β release was triggered by GBS hemolysin and required components of the canonical inflammasome, including caspase-1, ASC and NLRP3. Collectively our findings indicate that neutrophils make a significant contribution to IL-1β production during GBS infection, thereby amplifying their own recruitment. These cells directly recognize GBS by means of endosomal TLRs and cytosolic sensors, leading to activation of the caspase-1 inflammasome.

## Introduction

Interleukin-1β (IL-1β) is a well-characterized member of the IL-1 family of cytokines, which play important roles in the initiation of innate and adaptive immune responses. IL-1β acts as a regulator of tissue homeostasis by affecting several cell functions, including proliferation, differentiation and apoptosis [1, 2]. This cytokine orchestrates systemic and local inflammatory responses to noxious stimuli of any kind, thereby promoting host defenses. IL-1β is produced and secreted by a wide variety of cell types, including phagocytes, natural killer cells, keratinocytes and endothelial cells [1, 2]. A two-step mechanism is required for release of the cytokine. IL-1β is first translated as an inactive 31 kDa precursor (pro-IL-1β) in response to activation of specific receptors of the innate immune system, particularly Toll-like-receptors (TLRs). Next, pro-IL-1β is cleaved into its mature 17 kDa form and is released extracellularly after the activation of cysteine-aspartic proteases (caspases), particularly caspase-1, or by means of other proteolytic enzymes, such as serine proteases. Upon secretion into the extracellular space, IL-1β signals via the IL-1 receptor 1 (IL-1R1), which is expressed on many different cell types, including stromal cells, endothelial cells and bone marrow-derived resident and recruited immune cells [3].

Group B *Streptococcus* (GBS or *Streptococcus agalactiae*) is an encapsulated gram positive bacterium that can behave in the human host both as a harmless commensal of mucosal surfaces and as an invasive pathogen. GBS is a leading cause of serious infections in neonates, post-partum women, elderly people and in patients with underlying conditions, such as liver cirrhosis, diabetes and malignancies [4]. Over the years, this organism has been frequently used to model the innate immune responses and the pathophysiological manifestations occurring during invasive infections and sepsis caused by extracellular bacterial pathogens [5–10]. Innate immune recognition of GBS triggers the activation of a comprehensive cytokine program, which ultimately results in potentiation of phagocytic killing and clearance of bacteria [10–14]. In the context of anti-GBS defenses, a crucial role is played by primary cytokines, including TNF-α, IL-12 and interferon β, which are directly produced by the cells of the innate immune system after recognition of these bacteria [9, 11, 13, 15]. Moreover, recent studies point to the importance of a cytokine circuit initiated by IL-1β, also a primary cytokine, which leads to the recruitment of neutrophils through the induction of the CXCL1 and CXCL2 chemokines [6, 16]. GBS-stimulated IL-1β release has been studied only in dendritic cells and in macrophages [17]. In these cells, pro-IL-1β is first produced by a mechanism requiring phagocytosis, phagolysosomal processing [17] and activation of selected endosomal TLRs, including TLR7, TLR9 and TLR13 [7]. Little pro-IL1β is processed in the absence of a second signal, which is provided by hemolysin, a cytotoxin produced by GBS [7]. It is presently unclear how GBS β-hemolysin triggers processing of pro-IL-β. It has been proposed that, in macrophages, the function of hemolysin is to damage the phagolysosome membrane, leading to the escape of bacterial RNA into the cytosol [18]. Bacterial RNA would, in turn, bind to the cytosolic sensor NALP3, which activates the effector protease caspase-1 through the adaptor ASC [18]. In GBS-stimulated macrophages and dendritic cells, caspase-1 ultimately cleaves pro-IL-1β into the mature 17 kDa form and promotes its extracellular release [17, 18].

Little is known of GBS-induced IL-1β release in cells other than macrophages and dendritic cells. It is widely accepted that the mechanisms underlying pro-ILβ production and processing may vary considerably because of cell type-specific expression of recognition receptors and proteases [19–22]. In the present study, after finding that neutrophils make a significant contribution to IL-1β production during infection, we observed that live GBS induce in these cells activation of endosomal TLRs and of the caspase-1 inflammasome, leading to the release of mature IL-1β. Since IL-1β has a crucial role in attracting neutrophils to infection sites [16], our present data suggest that, after recognizing GBS, these phagocytes can amplify their own recruitment through IL-1β production.

## Results

### Neutrophils are the predominant IL-1β-producing cell type in GBS-induced exudates early during infection

**Fig 1:**
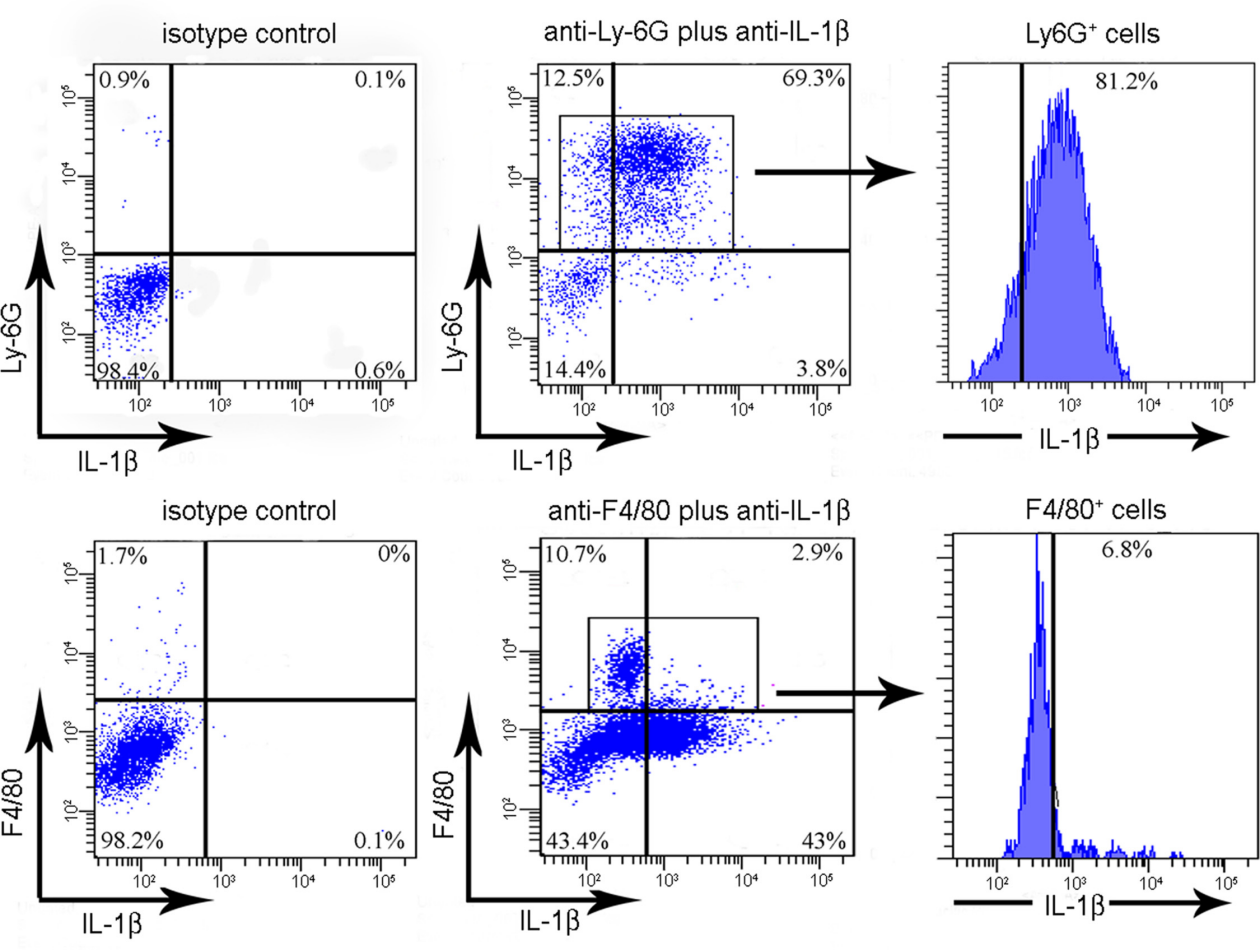
Neutrophils are the predominant cell type expressing IL-1β in response to challenge with GBS. Flow cytometry analysis showing cells positive for intracellular IL-1β staining in peritoneal lavage fluid samples from WT C57BL/6 mice challenged i.p. with HK-GBS. Neutrophils and macrophages were identified based on expression of Ly6G and F4/80, respectively. Data are from one representative experiment of three producing similar results.

Many different cell types, including resident and inflammatory cells, can produce IL-1β during infection. To gain insight into the cell types responsible for GBS-induced IL-1β release, we used a previously described murine peritonitis model [6, 16] whereby cell influx and cytokine production can be followed after the i.p. injection of heat-killed GBS (HK-GBS). To identify IL-1β-secreting cells in the exudates, intracellular IL-1β was visualized by direct immunofluorescence using PE-conjugated polyclonal anti-IL-1β antibodies and flow cytometry. We found that neutrophils and macrophages accounted for approximately 82% and 7%, respectively, of the IL-1β producing cells at 6 h after challenge with HK-GBS (Fig 1). Moreover, about 70% of Ly6G^+^ neutrophils in peritoneal exudates were IL-1β-positive (Fig 1). These data suggested that neutrophils might represent the most abundant cell type expressing IL-1β in response to GBS early during infection.

### Neutrophil depletion impairs GBS-induced IL-1β release

**Fig 2:**
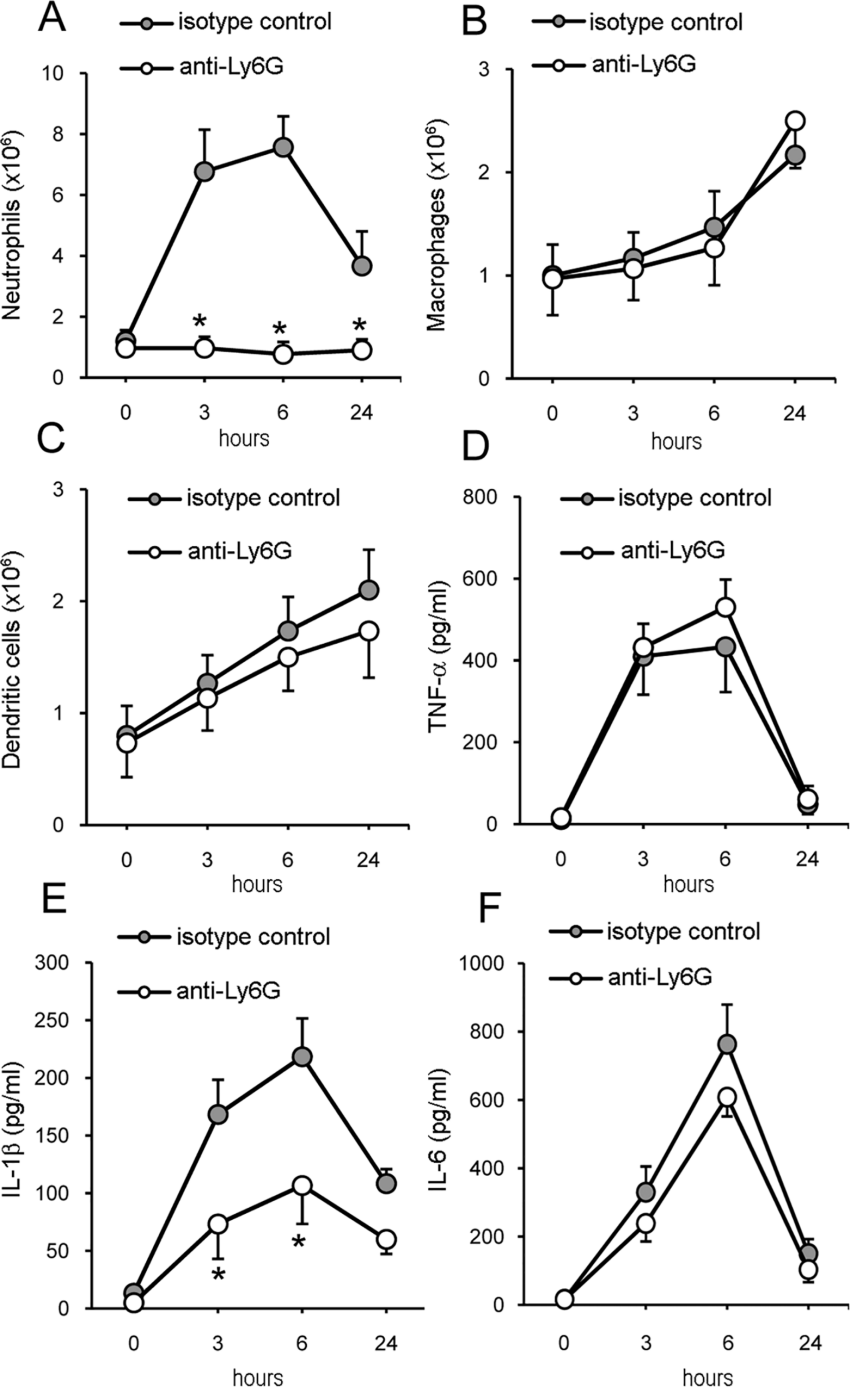
Effects of neutrophil depletion on cytokine production in response to GBS. WT C57BL/6 mice were pretreated with rat anti-Ly6G monoclonal antibody or isotype control Ig before i.p. challenge with HK-GBS. **A-C,** numbers of peritoneal cells positive for Ly6G (granulocytes), F4/80 (macrophages) and CD11c (dendritic cells) at the indicated times after HK-GBS challenge. **D-F**, Cytokine concentrations in peritoneal lavage fluid samples at the indicated times after HK-GBS challenge. Data are expressed as means±SD of three independent observations, each conducted on a different animal. *, *p*<0.05, relative to isotype control-pretreated mice by one-way analysis of variance and the Student’s-Keuls-Newman test.

To estimate the relative contribution of neutrophils to IL-1β production, we next examined the effects of neutrophil depletion on *in vivo* release of the cytokine. Wild type (WT) animals were injected with a rat monoclonal antibody (anti-Ly-6G clone 1A8), which is highly specific for neutrophils [23], or with an equal amount of isotype control Ig. Anti-Ly6G treatment was sufficient to reduce neutrophil blood counts to < 1% by 24 h and these low counts persisted for at least 72 h after treatment (S1 Table). Next, anti-Ly6G- or control Ig-treated mice were inoculated i.p with HK-GBS and cell influx and cytokine concentrations were measured in peritoneal lavage fluid samples. In control Ig-treated mice, total cell counts rapidly increased after GBS challenge, to reach peak levels at 3–6 h and decline thereafter (Fig 2). Cell influx was almost completely accounted for by neutrophils and a modest increase in macrophages was observed only at 24 h after challenge (Fig 2A–2C). As expected, neutrophils were almost completely absent in peritoneal exudates from anti-Ly6G-treated mice after GBS challenge, while macrophage or dendritic cell counts were similar to those of control mice (Fig 2A–2C). We next measured in these exudates the levels of IL-1β and of other proinflammatory cytokines known to be produced by neutrophils, such as TNF-α and IL-6 [24]. Cytokine elevations displayed the same kinetics as those of neutrophil influx, peaking at 3–6 h after GBS challenge and declining at 24 h. However, the levels of IL-1β, but not those of TNF-α and IL-6, were significantly reduced in neutrophil-depleted mice (Fig 2D–2F). Next we sought to confirm these data using live, instead of heat-killed GBS as a challenge. To this end, we inoculated mice i.p. with live organisms followed, after 1 h, by administration of penicillin to prevent bacterial overgrowth. It should be noted that, in this experimental setting, exposure to live bacteria was transitory, which likely reduced the extent and duration of the inflammatory response. Nevertheless, also under these conditions, the levels of IL-1β, but not those of TNF-α, were significantly reduced in the neutrophil-depleted mice (S1 Fig). Collectively, these data indicated that neutrophil depletion can result in a selective reduction of IL-1β levels in GBS-induced inflammatory exudates.

### Neutrophils directly produce IL-1β in response to stimulation with GBS

**Fig 3:**
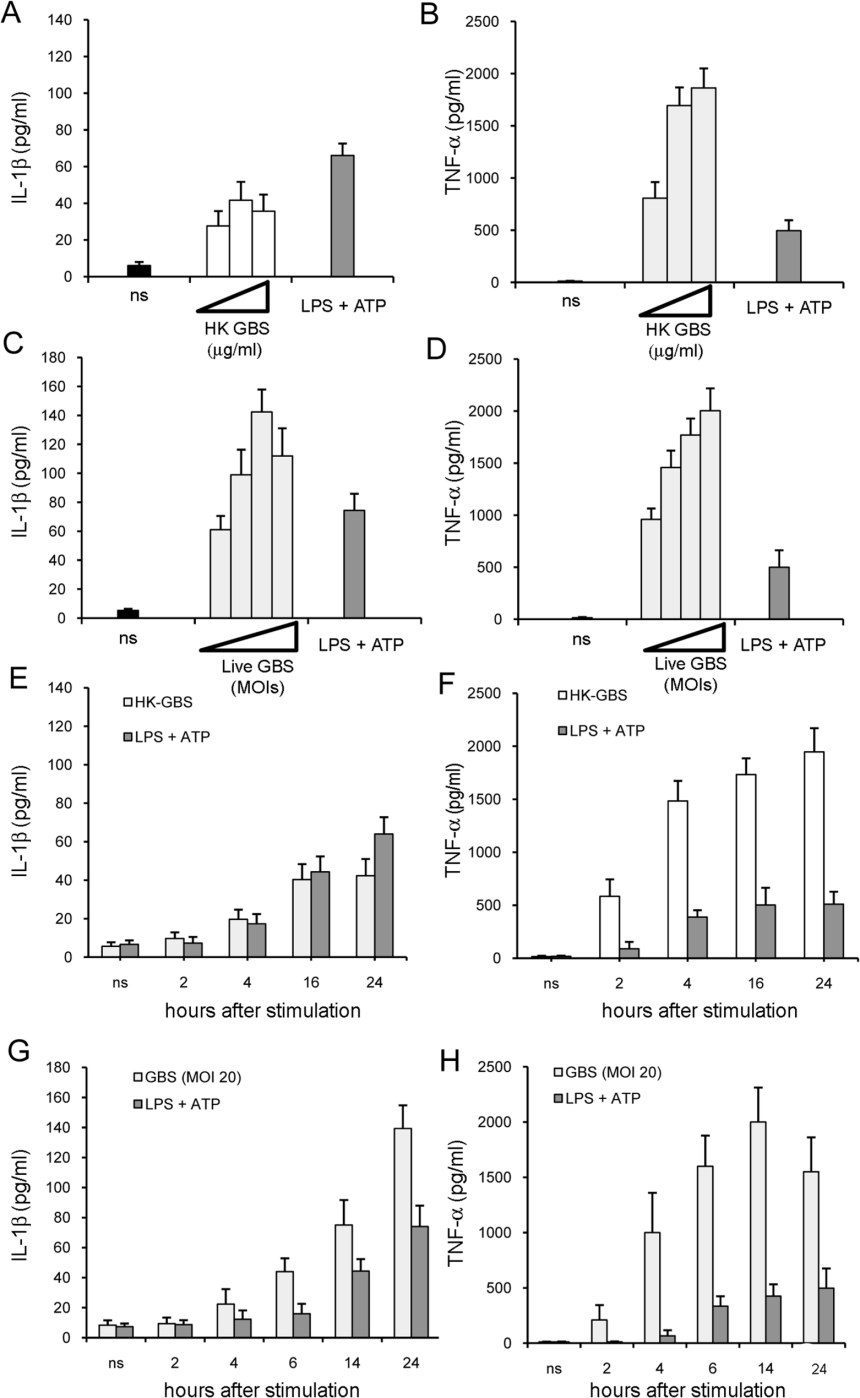
Release of IL-1β and TNF-α in neutrophil cultures stimulated with GBS. IL-1β and TNF-α concentrations in culture supernatants of neutrophils isolated from the bone marrow of WT C57BL/6 mice. Cytokines were measured at 24 h after treatment with increasing doses (1, 10 or 20 μg/ml) of HK-GBS (**A** and **B**) or increasing MOIs (5, 10, 20 or 40) of live GBS (**C** and **D**). Kinetics of IL-1β (**E** and **G**) and TNF-α (**F** and **H**) production in neutrophils stimulated with HK-GBS (10μg/ml) or with live GBS (MOI of 20). Neutrophils treated with LPS (0.1 μg/ml), and then pulsed with ATP (5mM) for 30 min before collecting supernatants, served as controls. Data are expressed as means+SD of three independent experiments.

Although the data presented above indicated that neutrophils are capable of producing IL-1β during GBS infection, they could not evidence whether such production occurs by direct or indirect mechanisms. To investigate this, bone-marrow-derived neutrophil preparations were exposed *in vitro* to various doses of GBS. We initially used HK-GBS as stimuli and, as a positive control, LPS pre-treatment, followed by the addition of ATP, an activator of the NALP-3 inflammasome [25–27]. HK-GBS stimulated modest IL-1β release (Fig 3A), while inducing high levels of TNF-α (Fig 3B). However, when we used live bacteria as stimulus, robust IL-1β responses were also observed (Fig 3C). Moreover, TNF-α and IL-1β release was dose-dependent after stimulation with live bacteria (Fig 3C and 3D). Significant elevations were not detected before 4h after the beginning of stimulation, suggesting that cytokine release required *de novo* synthesis (Fig 3E–3H). Cytokine release was not the result of cell lysis, since exposure to live GBS at an optimal stimulating dose was not associated with decreased cell viability or increased apoptosis, as compared with unstimulated cultures (S2 Fig). Finally, cytokine production could not be accounted for by contamination of neutrophil preparations with macrophages, since removal of these cells with anti-CD115 antibodies coupled to magnetic beads did not affect IL-1β production (S2 Table and S3 Fig).

**Fig 4:**
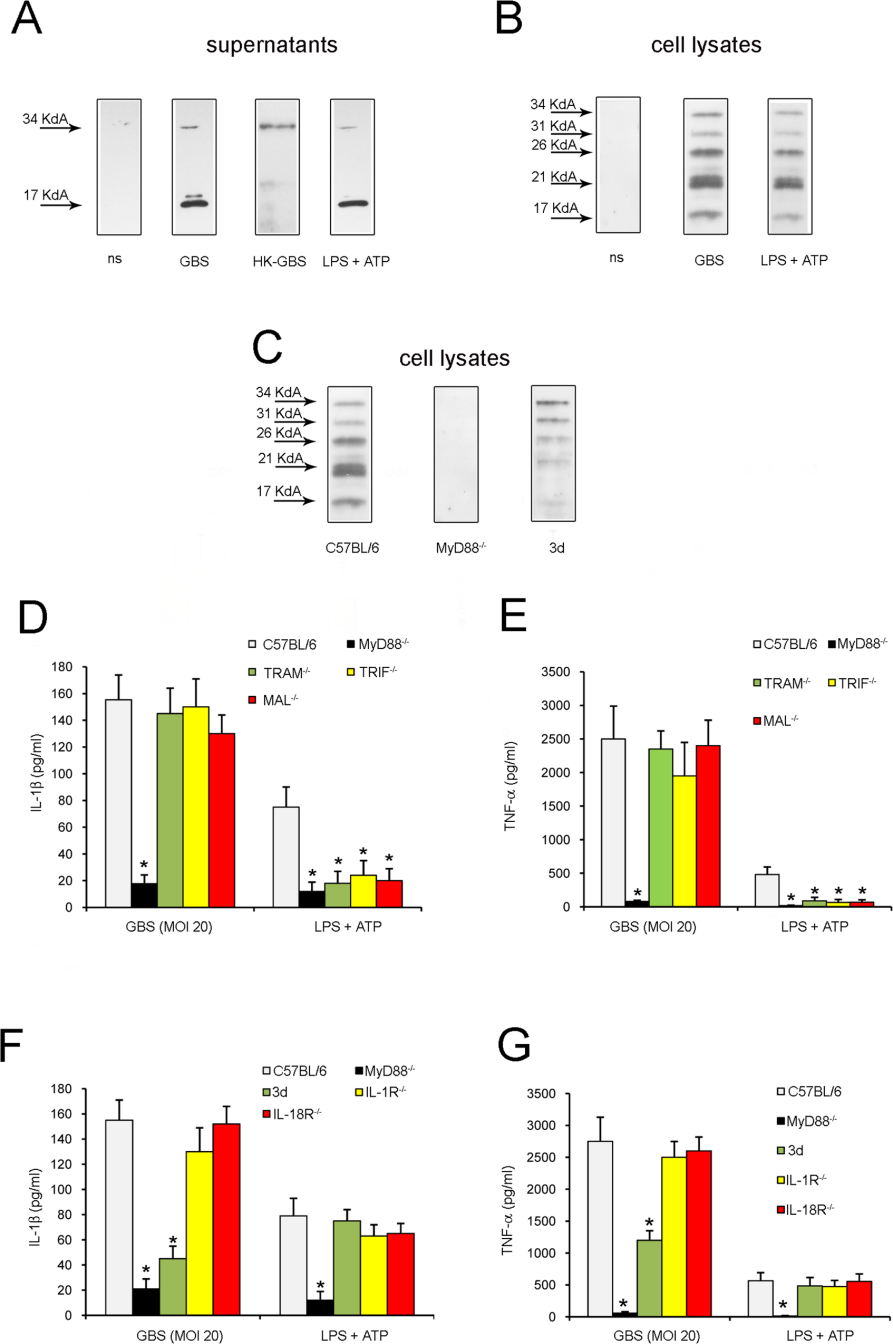
GBS-induced pro-IL-1β production is dependent on MyD88 and multiple endosomal TLRs. Western blot analysis of precipitated supernatants (**A**) or cell lysates (**B and C**) obtained from cultures of WT neutrophils treated with live (MOI 20) or HK-GBS (10 μg/ml). Immunoreactive bands were detected using anti-IL-1β. ns, non-stimulated samples. Concentrations of IL-1β (**D** and **F**) and TNF-α (**E** and **G**) in supernatants of neutrophils lacking the indicated gene products involved in TLR or cytokine signaling. 3d, neutrophils from 3d mutant mice lacking functional UNC93B1. Supernatants were collected at 24 h after infection with live GBS (MOI 20). The positive controls consisted of neutrophils treated with LPS (0.1μg/ml) and then pulsed with ATP (5mM) for 30 min before collecting supernatants. Data are expressed as means + SD of three independent observations, each conducted with cells from a different animal. *, *p*<0.05 versus WT mice, as determined by one-way analysis of variance and the Student's–Keuls–Newman test.

Immunoreactive IL-1β was mostly represented by the 17 kDa mature form in the supernatants of neutrophil cultures stimulated with live GBS, although small quantities of the inactive precursor (pro-IL1β) were also detected, as indicated by western blot analysis (Fig 4A). In contrast, most of the immunoreactive IL-1β was represented by pro-IL1β in the supernatants of HK-GBS-stimulated neutrophils. Moreover, western blot analysis of neutrophil lysates revealed the intracellular presence of processed forms of pro-IL-1β (migrating with an apparent molecular weight of approximately 26, 21 and 17 kDa) after stimulation with either live GBS or LPS plus ATP (Fig 4B). Collectively, this set of data indicated that live GBS can directly stimulate neutrophils to produce and process pro-IL-1β, resulting in extracellular release of the mature cytokine.

### Induction of IL-1β responses requires endosomal TLRs

**Fig 5:**
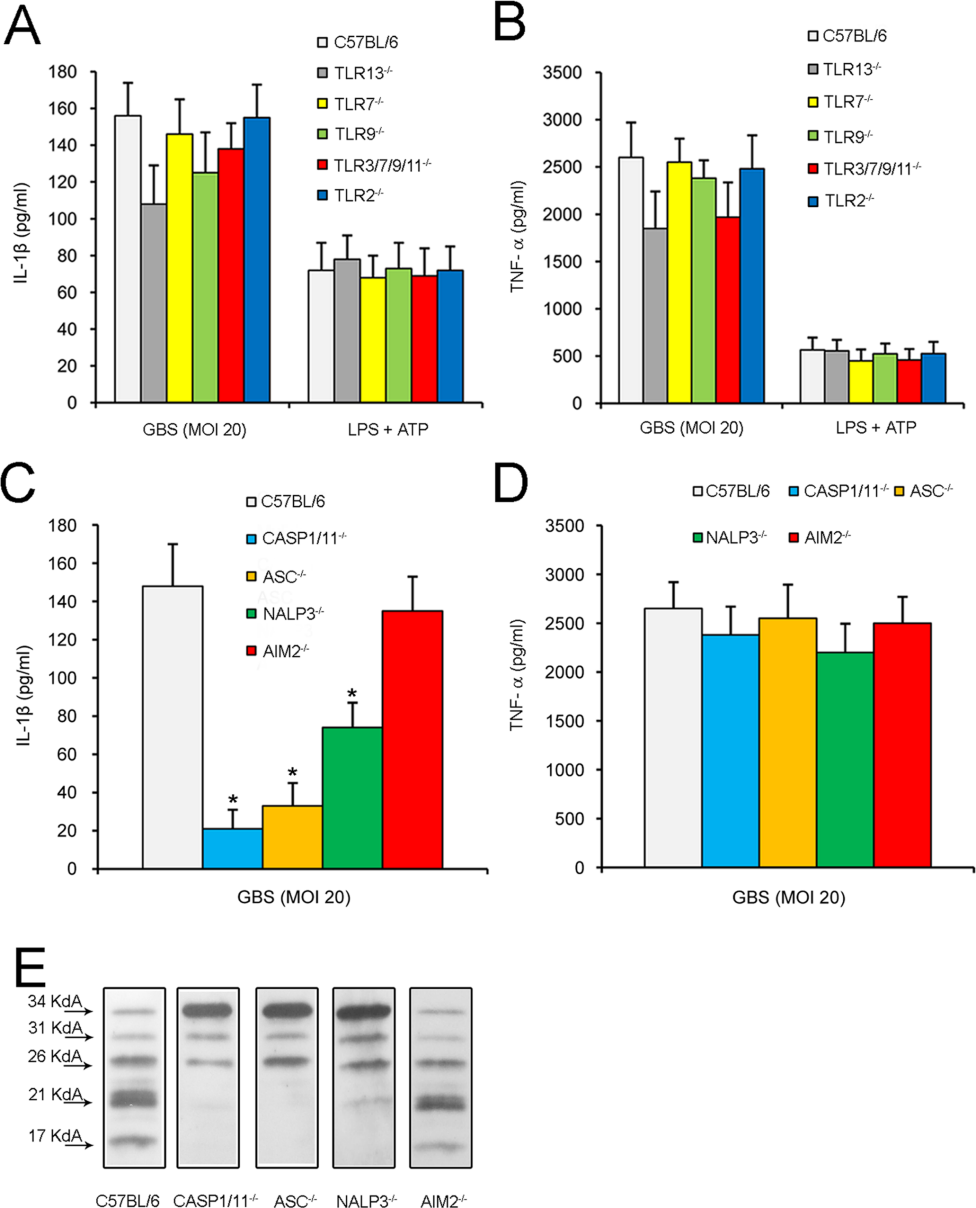
The caspase 1 inflammasome is involved in pro-IL-1β processing and IL-1β release in GBS-infected neutrophils. Concentrations of IL-1β (**A** and **C**) or TNF-α (**B** and **D**) in culture supernatants of neutrophils lacking the indicated TLRs or inflammasome components. Supernatants were collected at 24 h after infection with live bacteria (MOI 20). Positive controls consisted of cells treated with LPS (0.1μg/ml) and then pulsed with ATP (5mM) for 30 min before collecting the supernatants. Data are expressed as means + SD of three independent observations, each conducted with cells from a different animal. *, *p*<0.05 versus WT mice, as determined by one-way analysis of variance and the Student's-Keuls-Newman test. **E**, Western blot analysis, using anti-IL-1β antibodies, of lysates from neutrophils lacking the indicated inflammasome components. Neutrophils were infected with GBS (MOI 20) for 4h.

Since previous studies in dendritic cells have shown that GBS-induced pro-IL-1β production requires TLR-mediated recognition of these bacteria [17, 18], we sought to investigate whether similar requirements applied to neutrophils. To this end, we used neutrophils isolated from the bone marrow of mice with genetic defects in TLR adaptor/chaperone proteins or in single TLRs. Release of both IL-1β (Fig 4D) and TNF-α (Fig 4E) after stimulation with live bacteria required MyD88, a TLR adaptor protein, but not other TLR adaptors, such as MAL, TRIF or TRAM [28, 29]. Failure to release IL-1β in MyD88-deficient neutrophils was accounted for by defective production of pro-IL-1β, as evidenced by western blot analysis of cell lysates (Fig 4C). Since MyD88 is involved in the transduction of signals originating not only from TLRs, but also from IL-1R and IL-18R, in further experiments we stimulated cells lacking IL-1R or IL-18R to ascertain whether IL-1/18 signaling might be involved in GBS-induced cytokine production. However, cell lacking IL-1R or IL-18R were fully capable of responding to GBS, indicating that the requirement for MyD88 was linked to TLR signaling (Fig 4F and 4G). We next tested cells from 3d mice, which have defective signaling of endosomal TLRs, such as TLR 3/7/9/13, due to a mutation in UNC93B1, a chaperone protein required for the localization of TLRs to endosomal compartments [30]. Neutrophils from 3d mice were impaired in their ability to produce pro-IL-1β (4C) and to release IL-1β (Fig 4F) or TNF-α (Fig 4G). In contrast, lack of single endosomal TLRs or of TLR2, a receptor involved in sensing of GBS lipoproteins [31], did not significantly impair IL-1β or TNF-α release (Fig 5A and 5B). There was a tendency for neutrophils lacking TLR13 to produce lower amounts of both cytokines, but these differences were not statistically significant. In addition, no defect was observed in cells simultaneously lacking the endosomal TLRs 3/7/9/11. All together, these data suggest that pro-IL-1β and TNF-α production in neutrophils requires MyD88-dependent transduction of signals originating from the simultaneous activation of multiple endosomal TLRs.

### Caspase-1 is required for IL-1β release in GBS-stimulated neutrophils

**Fig 6:**
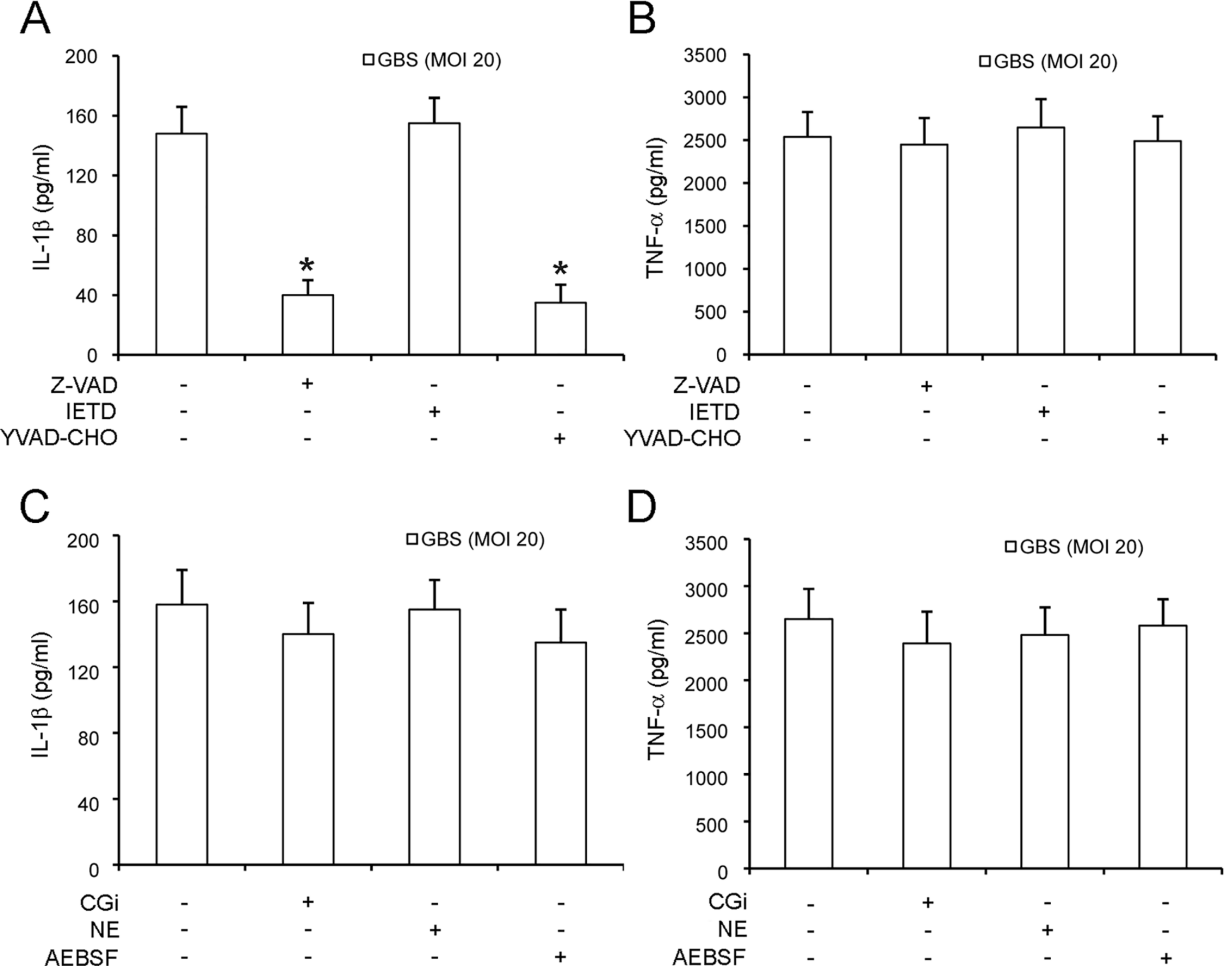
IL-1β processing by neutrophils is mediated by caspase-1. Mouse neutrophils (5 x 10^5^/well) were incubated for 1 h in the presence of Z-VAD, YVAD-CHO, IETD, AEBSF, NE (all at a concentration of 10μM), or CGi (0.5μM). Live GBS (MOI 20) were added and secreted IL-1β (**A, C**) and TNF-α (**B, D**) were measured in culture supernatants after 24 h of incubation. Data are expressed as means + SD of three independent observations, each conducted with cells from a different animal. *, *p*<0.05 versus untreated cells, as determined by one-way analysis of variance and the Student's–Keuls–Newman test.

Extracellular release of mature IL-1β requires processing of pro-IL-1β by caspases, such as caspase-1 and caspase-8, or by other proteolytic enzymes. To investigate if caspase-1 is required for GBS-induced IL-1β release, we used neutrophils from mice lacking this enzyme as well as caspase-11 (caspase-1/11^-/-^). Fig 5C shows that marked reductions in IL-1β levels were measured in the supernatants of GBS-stimulated neutrophils lacking caspase-1/11. We also examined the role of cytosolic sensors or adaptors, such as NALP3, ASC and AIM2, which participate in the formation of the inflammasome, a multi-protein complex involved in caspase-1 activation [32]. To this end, we stimulated NALP3-, ASC- or AIM2-deficient neutrophils with GBS and then analyzed the secretion of IL-1β. We observed that IL-1β secretion was at least partially dependent on NALP3 and ASC, but not on AIM2, as revealed by significant reductions of IL-1β secretion in NALP3- or ASC-deficient neutrophils (Fig 5C). As expected, caspase-1/11, NALP3 and ASC were dispensable for the production of TNF-α in response to GBS (Fig 5D). In further experiments, we investigated whether caspase-1, NALP3, ASC or AIM2 are involved in pro-IL-1β processing using western blot analysis of cell lysates from GBS-stimulated neutrophils. As shown in Fig 5E, IL-β processing was reduced in cells lacking caspase-1/11, ASC or NALP3. Next we sought to obtain additional insights into the caspase-1/11 requirement by using YVAD-CHO (a selective caspase-1 inhibitor), IETD (a selective caspase-8 inhibitor) and the pan-caspase inhibitor Z-VAD. Since neutrophil serine proteases, such as cathepsin G and elastase, also have the ability to process pro-IL-β, we used, in addition, the broad-spectrum serine protease inhibitor AEBSF and selective inhibitors of cathepsin G (CGi) and neutrophil elastase (Elastase inhibitor IV or NE). Fig 6 shows that only YVAD-CHO and Z-VAD, but not IETD or any of the serine protease inhibitors, significantly reduced IL-1β release. Collectively, these results suggest that infection with GBS triggers, in neutrophils, pro-IL-β processing and IL-β secretion via a mechanism that largely depends on caspase-1, but not on caspase-8 or on serine proteases.

### GBS hemolysin promotes IL-1β release

**Fig 7:**
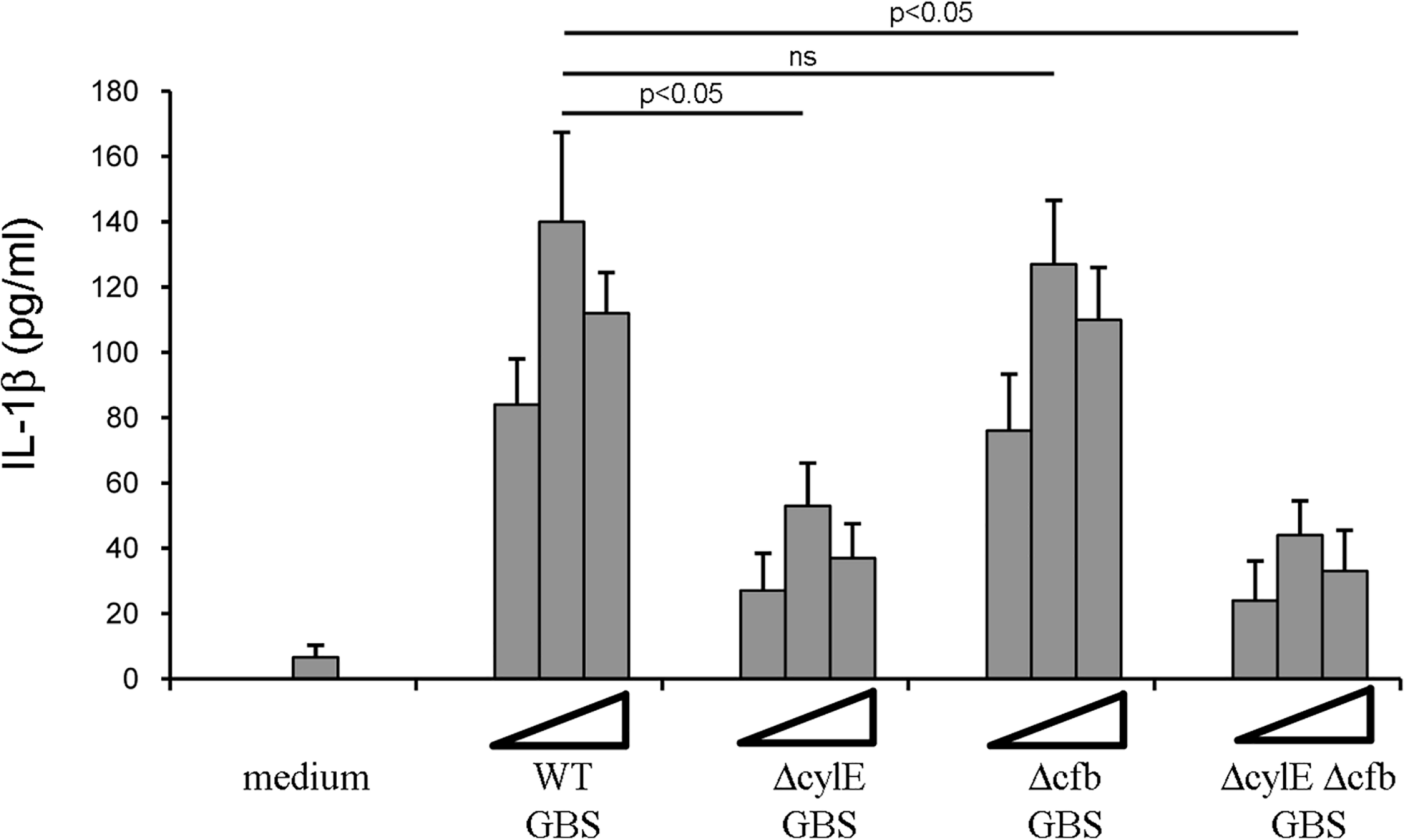
GBS β-hemolysin, but not CAMP factor, is involved IL-1β release. Concentrations of IL-1β in supernatants of WT neutrophil cultures collected at 24 h after infection with live GBS (MOI 20). WT strain NEM316 or its isogenic mutants deficient in β-hemolysin (ΔcylE), CAMP factor (Δcfb), or both (ΔcylE Δcfb) were used for stimulation. Data are expressed as means + SD of three independent observations, each conducted with cells from a different animal.

Previous studies have demonstrated that, in dendritic cells, GBS β-hemolysin can activate the NALP3 inflammasome, resulting in the secretion of IL-1β [17]. Therefore, we sought to determine whether, also in neutrophils, GBS-induced IL-1β release is dependent on β-hemolysin. To this end, neutrophils were infected with GBS lacking β-hemolysin, CAMP-factor (an additional GBS cytolysin) or both. Strains lacking β-hemolysin, but not CAMP-factor, were unable to induce high-level secretion of IL-1β in neutrophils (Fig 7). These data indicate that GBS β-hemolysin is required for IL-1β release in GBS-stimulated neutrophils.

## Discussion

The innate immune system is extremely effective in controlling GBS replication and most individuals who are colonized with these bacteria do not develop infection even when lacking anti-GBS antibodies [33, 34]. In these innate defenses, a key role is played by IL-1β, which contributes to the release of secondary chemotactic mediators and promotes neutrophil mobilization from the bone marrow into sites of GBS replication [6, 16]. However, the main cell types contributing to IL-1β release during GBS infection have not been studied. Using models of GBS-induced peritoneal inflammation, we found here that locally recruited neutrophils make a significant contribution to IL-1β production, as evidenced by a predominance of neutrophils among the IL-1β-positive cells and by reduced IL-1β levels after neutrophil depletion. These findings may be clinically relevant, since neutropenic neonates have increased lethality rates following invasive GBS infections [35, 36], mirroring the extreme susceptibility to the pathogen of neutrophil-depleted mice [16]. It should be pointed out, however, that human and murine neutrophils differ in several aspects, including inflammasome functions [37], and that further studies using human cells are needed to analyze GBS-inflammasome interactions. Collectively, our data suggest that the protective activities of neutrophils may rest not only on their ability to kill GBS, but also on their capacity to release IL-1β. Traditionally, neutrophils have been assigned the role of short-lived effector cells with limited ability to influence the function of other cell types through mediator release. However, these cells can remain viable for longer periods of time than originally thought (particularly in exudates rich in proinflammatory cytokines and growth factors) and are an important source of pro-inflammatory and regulatory cytokines, such as IFN-γ and IL-17A [38–40]. Data presented here lend further support to this notion and extend recent observations on the importance of neutrophil-derived IL-1β in selected infections, such as *Staphylococcus aureus* skin abscesses [22] and *Pseudomonas aeruginosa* [21] or *Streptococcus pneumoniae* [41] keratitis. Together, our previous studies and the present one document the existence of a positive feedback loop whereby neutrophils recruited to infection sites promote further neutrophil influx by producing IL-1β [6, 16]. However, cell types other than neutrophils also participate in IL-1β release, since neutrophil depletion reduced, but did not abrogate, IL-1β release after GBS challenge. Since macrophages can release IL-1β in response to GBS [17], it is likely that they also play a role in the production of this cytokine, particularly after 24 h from the beginning of infection, when these cells start to be efficiently recruited to the infection site [16].

In the second part of our studies, we focused on the mechanisms leading to IL-1β production after direct recognition of GBS by neutrophils. Little is known of the production of this, or other cytokines, in neutrophils. An early study described IL-8 release following stimulation of human polymorphonuclear leukocytes with heat-killed GBS, but the receptors involved or the production of other cytokines were not investigated [42]. Using IL-1β and TNF-α release as a read-out for anti-GBS responses, we found that neutrophils recognize these bacteria by mechanisms involving endosomal TLRs, as suggested by: 1) the complete dependence of anti-GBS responses on the TLR adaptor MyD88, in conjunction with lack of involvement of non-TLR receptors also signaling through MyD88, such as IL-1R or IL-18R; 2) the partial dependence on UNC93B1, a chaperone protein involved in signaling by endosomal TLRs [30, 33]. Together with previous studies showing the ability of GBS mRNA, rRNA and DNA to stimulate, respectively, TLR7, TLR13 and TLR9 [7, 15], these data suggest that pro-inflammatory cytokine production in neutrophils depends at least in part on the concerted action of these three receptors, which are all known to be UNC93B1-dependent. Notably, lack of individual TLRs had no effect on cytokine induction, suggesting that the absence of a single receptor can be compensated for by the others. Collectively our data indicate that neutrophils can drive pro-inflammatory responses after GBS recognition by mechanisms that resemble those of macrophages and dendritic cells [5, 15, 17].

An important step in the release of mature IL-1β is proteolytic cleavage of pro-IL-1β, a process that can be mediated by activation of some caspases [43]. However, especially in neutrophils, serine-proteases (such as proteinase 3, neutrophil elastase or cathepsin G) can also process pro-IL-1β, allowing the release of mature, biologically active forms [44, 45]. The relative importance of the various proteolytic mechanisms varies in different cell types and models of inflammation and infection [19]. For example, caspase 1-independent IL-1β substantially contributes to host defenses against *Mycobacterium tuberculosis* [20], *Pseudomonas aeruginosa* [21] or to sterile inflammation induced by silica [46] and diesel exhaust [47] particles. In view of the cell-type specificity in the mechanisms underlying IL-1β release, we studied in detail pro-IL1β processing in GBS-stimulated neutrophils. Our data using genetically defective cells and specific inhibitors indicate that GBS-induced IL-1β release requires functional caspase 1 and is significantly reduced by lack of NALP3 or ASC, which are well-characterized components of the canonical caspase-1 inflammasome. These findings extend those of previous reports implicating NALP3/ASC/caspase-1 in neutrophil-dependent IL-1β secretion induced by *S*. *aureus* [22] and *S*. *pneumoniae* [41].

In conclusion, the results of the present study suggest that the role of neutrophils during GBS infection is more sophisticated than originally thought and is not confined to pathogen killing. These cells can recognize GBS as efficiently as mononuclear phagocytes using a range of endosomal TLRs and can produce significant amounts of proinflammatory cytokines, thereby orchestrating further influx of neutrophils and other cell types.

## Materials and Methods

### Mice

Caspase-1/11^−/−^ mice were obtained from Arturo Zychlinski (Max Planck Institute, Berlin, Germany). ASC^−/−^, NALP3^−/−^ and AIM2^-/-^ animals were obtained from Vishva Dixit (Genentech, CA). Mice lacking single TLRs (TLR 2, 3, 7 or 9), multiple TLRs (TLR 3/7/9/11) or TLR adaptors (MyD88, TRAM, TRIF or MAL) were developed as described [48] and obtained from Shizuo Akira (Osaka University, Japan). 3d mutant mice, bearing the H412R mutation in the chaperone protein UNC93B1, were obtained from Bruce Beutler (University of Texas Southwestern Medical Center, TX). TLR13^-/-^ mice originated from the embryonic stem cell clone 10438B-B6, produced by Regeneron Pharmaceuticals, Inc. using previously described methods [7]. Heterozygous TLR13^-/+^ mice were provided by the KOMP Repository (www.komp.org) and the Mouse Biology Program (www.mousebiology.org) at the University of California Davis. TLR13^-/-^ mice, bred on a C57BL/6J background, were born and developed normally in the animal facilities of the Department of Human Pathology of the University of Messina, Messina, Italy. C57BL/6 wild-type (WT) mice, used as controls, were purchased from Charles River Laboratories. All mice used in the present study were housed under specific pathogen-free conditions in individually ventilated cages.

### Reagents

The following inhibitors were purchased from Calbiochem: Z-VAD (pan-caspase inhibitor), YVAD-CHO (selective caspase-1 inhibitor), IETD (selective caspase 8 inhibitor), CGi (selective cathepsin G inhibitor), AEBSF (broad spectrum serine protease inhibitor) and elastase inhibitor IV (NE, a selective neutrophil elastase inhibitor). ATP was purchased from Sigma-Aldrich. Ultra pure LPS was from InvivoGen.

### GBS strains and murine peritonitis models

GBS WT strain H36B serotype Ib was used for most experiments. In selected experiments we also used the NEM316 strain and its isogenic mutants deficient in β-hemolysin (ΔcylE), CAMP factor (Δcfb), or both (ΔcylE Δcfb), which have been previously described [17]. GBS were grown to the mid-log phase in Todd-Hewitt broth (Oxoid), washed twice in nonpyrogenic PBS (0.01 M phosphate, 0.15 M NaCl [pH 7.4]; Euroclone), and resuspended to the desired concentration in PBS. Lyophilized heat-killed GBS (HK-GBS) was prepared as previously described [16, 49].

Six-wk-old female mice were injected i.p. with HK-GBS (1 mg/mouse) in PBS (0.2 ml), and peritoneal lavage fluid was collected at various times to measure cell numbers by flow cytometry and cytokine concentrations by ELISA (see below), as previously described [14, 15, 50]. In some experiments mice were injected i.p. with live H36B GBS grown to the mid-log phase (2x10^7^ CFU in 0.2 ml of PBS) and treated 1 h later with penicillin (0.5 mg i.p) to prevent bacterial overgrowth. In selected experiments, WT mice were pretreated i.v. with 100 μg of rat monoclonal anti-mouse Ly6G antibody (clone 1A8) or rat IgG2a control (isotype control; both from BD Pharmingen) 24 h before i.p. challenge with live or heat-killed GBS, as previously described [16].

### Isolation and stimulation of neutrophils

Neutrophils were obtained from the bone marrow of WT and KO mice using Percoll density gradient centrifugation, as previously described [51]. Briefly, after removing the femurs and the tibias, RPMI 1640 containing 10% (v/v) fetal calf serum (FCS) was forced through the bone with a syringe. For isolation of neutrophils, bone marrow cells were layered on the top of a 62 and 81%, two-layer discontinuous Percoll (GE Healthcare Life Sciences) gradient, as described [51]. After centrifugation at 1060 x g for 30 min at room temperature, neutrophils were harvested from the interface of the two layers, washed extensively in PBS to remove Percoll, and re-suspended in RPMI containing 10% (v/v) FCS. The viability of cells obtained *via* this procedure was routinely > 90% as assessed by trypan blue exclusion assay. Isolated cells (5 ± 0.6 × 10^6^ cells per mouse) were stained with May/Grunwald/Giemsa [52], and approximately 90% of them were morphologically mature neutrophils (bands and segmented). Purity of neutrophil populations was also determined by flow cytometry using the neutrophil-specific marker Ly6G (see below). In selected experiments Percoll-isolated cells were further treated with the anti-CD115 MACS bead separation kit (Miltenyi Biotec) to remove contaminating macrophages, as described [22].

Isolated neutrophils (5 x 10^5^ per well in 0.2 ml of RPMI supplemented with 10% FCS) were seeded in microtiter plates and stimulated with HK or live GBS grown to the mid-log phase at various multiplicities of infection (MOI). All infections were carried out by centrifuging cell suspensions for 10 min at 400 x *g* in order to facilitate bacteria/neutrophil interactions. After incubation for 1 h at 37°C, penicillin (250 IU/ml) and streptomycin (250 μg/ml) were added to kill extracellular bacteria. In preliminary experiments we verified that this antibiotic treatment did not affect viability of intracellular bacteria, as shown by counting colony forming units in cell lysates. Positive control wells were stimulated with *Escherichia coli* K12 ultrapure LPS (100 ng/ml; InvivoGen) before the addition of ATP (5 mM; Sigma-Aldrich) for 30 min before collection of the supernatants. Cell culture supernatants or cell lysates were collected at various times after stimulation and stored at -80°C for cytokine measurements and immunoblot analysis. For the inhibitor studies, neutrophils (5 x 10^5^/well) were preincubated for 1 h at 37°C, with Z-VAD, YVAD-CHO, IETD, AEBSF, CGi or NE, at the indicated concentrations in culture medium with 5% dimethylsulphoxide (final concentration) before stimulation with live GBS. Control wells were treated with vehicle only.

### Cytokine measurements

IL-1β, IL-6 and TNF-α concentrations were determined in duplicate using the following murine enzyme-linked immunosorbent assay (ELISA) kits (all from R&D Systems): IL-1β/IL-F2 Quantikine; TNF-alpha Duoset; IL-6 Quantikine. The lower detection limits of these assays were, respectively, 8, 16 and 8 pg/ml. Cytokine measurements were performed according to the manufacturer’s recommendations, as previously described [49, 53].

### Immunoblotting

Pro-IL-1β and processed IL-1β forms were detected by immunoblotting, as previously described [17, 50, 54]. Briefly, supernatants were precipitated with 10% trichloroacetic acid (Sigma-Aldrich) and washed in cold acetone by centrifugation at 12,000 × *g* for 10 min at 4°C. Next, pellets were dried and resuspended in sample buffer. For analysis of cell lysates, the plates were incubated on ice for 10 min, and then the cells were detached with a scraper and transferred into 1.5-ml Eppendorf tubes. Cells were lysed with 50 μl of lysis buffer (50 mM Tris-HCl [pH 7.4], 100 mM NaCl, 1% Triton X-100, and 5% glycerol, all purchased from Sigma-Aldrich) supplemented with a mixture of protease inhibitors (Roche). The lysates were centrifuged at 12,000 × *g* for 10 min at 4°C, to eliminate cellular debris and proteins were separated on 15% polyacrylamide gels and transferred to nitrocellulose membranes (Bio-Rad). Immunoblotting was performed using goat anti-IL-1β (1:800; R&D Systems). Immunoblots were developed using HRP-conjugated secondary antibodies (1:15,000 R&D Systems) and developed with Luminata Forte Western HRP substrate (Biorad).

### Flow Cytometry

Flow cytometry analysis of leukocyte subsets in peritoneal lavage fluid was performed on a FACS Canto II flow cytometer (BD Biosciences) as previously described [6, 53]. Briefly, all cells were incubated with 0.5 μg Fc Block (BD Biosciences) for 20 minutes before staining for 20 minutes with antibodies directed against F4/80 (macrophages), Ly-6G (clone 1A8, which is highly specific for neutrophils [23]) or CD11c (dendritic cells) using the respective isotype Abs as controls. Cells count were determined in peritoneal lavage fluids using BD TruCount tubes, as previously described [6]. For intracellular IL-1β staining, cells were permeabilized with Cytofix/Cytoperm buffer (BD Biosciences) for 20 min and then stained with PE-conjugated anti-mouse IL-1β (eBioscience). Data were analyzed using the Flow Jo software (BD Biosciences). In selected experiments, percentage of apoptosis and dead cells were assessed with flow cytometry using the PE Annexin V Apoptosis Detection Kit (BD Pharmigens), following the manufacturer's instructions. Cells staining only for Annexin-V were considered to be in early apoptosis (“apoptotic cells”) while those staining for both Annexin-V and 7-Amino-Actinomycin (7-AAD; a vital dye) were considered to be in late apoptosis or necrosis (“dead cells”). Cells negative for Annexin-V/7-ADD were considered to be viable.

### Data expression and statistical significance

Differences in cytokine levels and cell counts were assessed by one-way analysis of variance and the Student's-Keuls-Newman test. When *p* values were lower than 0.05, differences were considered statistically significant.

### Ethics statement

All studies were performed in strict accordance with the European Union guidelines for the use of laboratory animals (Directive 2010/63/EU). The procedures were approved by the Animal Welfare Committee of the University of Messina (OPBA permit no. 18052010) and by the Ministero della Salute of Italy (permit no. 665/2015).

## Supporting Information

S1 Fig Effects of neutrophil depletion on cytokine production during GBS infection. (PDF)

S2 Fig Neutrophil viability and apoptosis after stimulation with GBS. (PDF)

S3 Fig Effect of removal of macrophages from Percoll-isolated bone marrow cells on IL-1β responses. (PDF)

S1 Table Effect of anti-Ly6G treatment on blood polymorphonuclear leukocytes counts. (PDF)

S2 Table Effect of removal of macrophages with anti CD115^+^-coated beads on purity of bone marrow-derived neutrophil preparations. (PDF)
