# Supplementary figures and images for: Neutrophils Directly Recognize Group B Streptococci and Contribute to Interleukin-1β Production during Infection
Source: PLoS One. 2016 Aug 10;11(8):e0160249. doi: 10.1371/journal.pone.0160249 (PMC4980021; doi:10.1371/journal.pone.0160249)

S1 Fig.

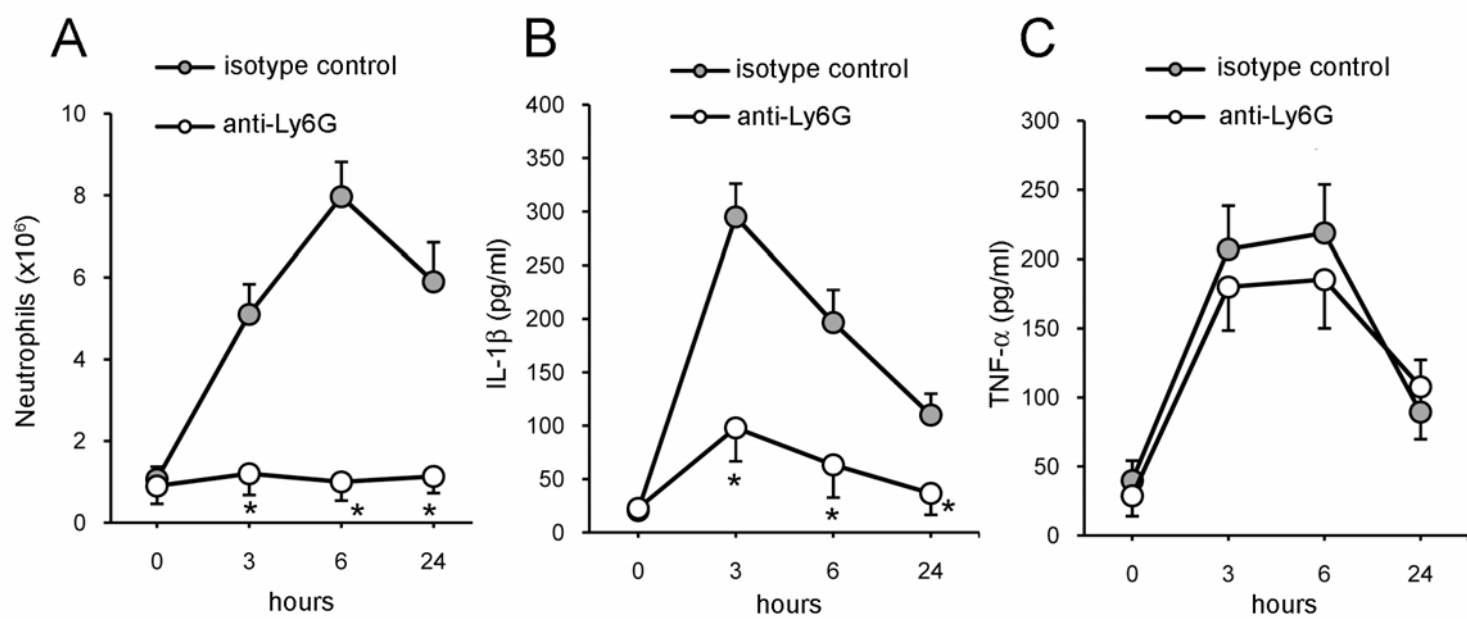

Supplement: S1 Fig — (PDF) [file pone.0160249.s001.pdf]

S2 Fig.

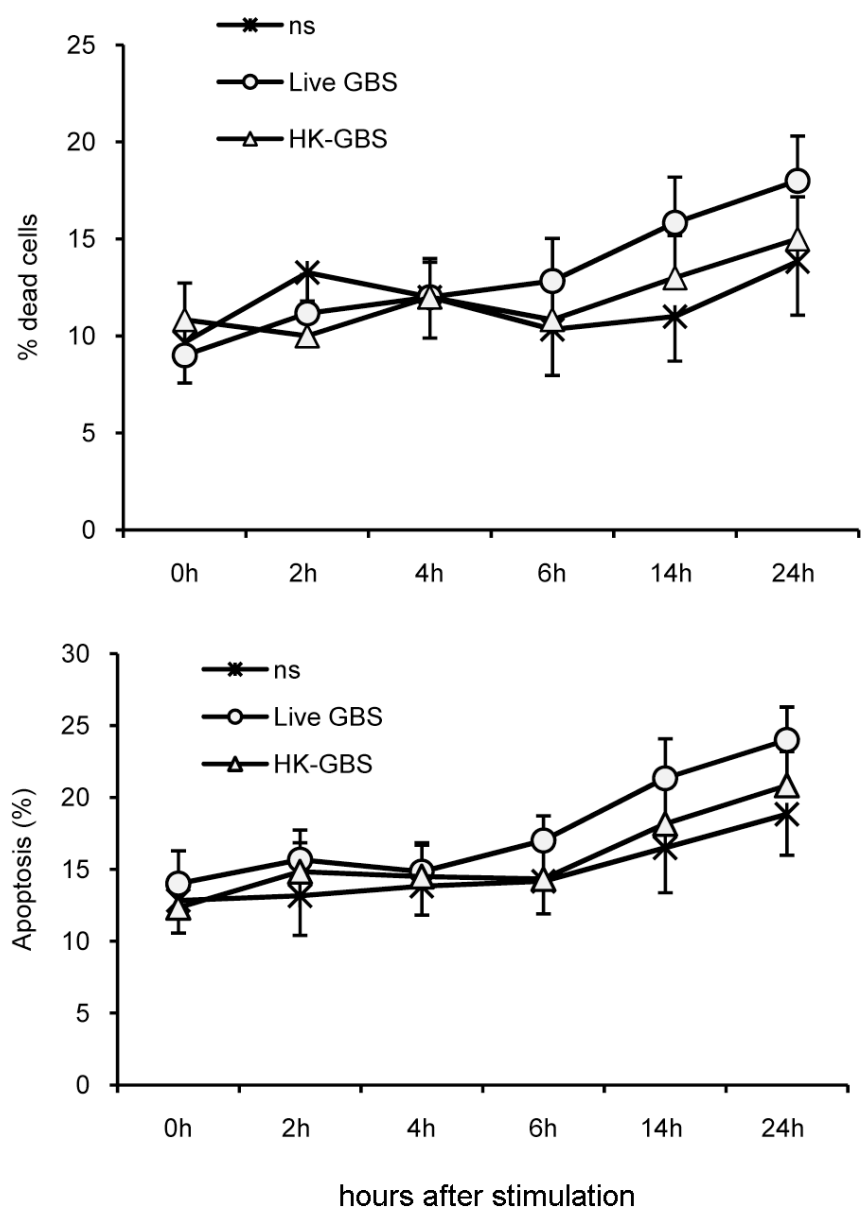

Supplement: S2 Fig — (PDF) [file pone.0160249.s002.pdf]

S3 Fig.

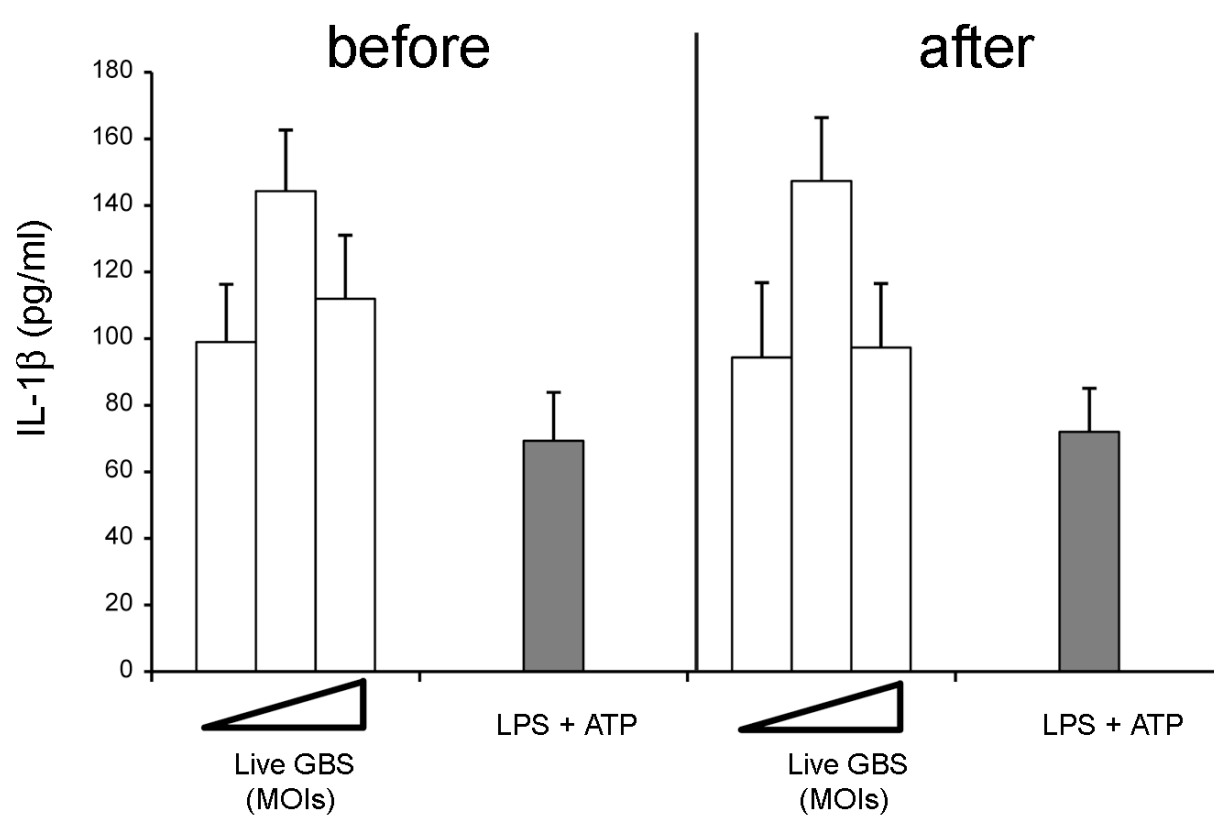

Supplement: S3 Fig — (PDF) [file pone.0160249.s003.pdf]
