# Supplementary material for: Neutrophils Directly Recognize Group B Streptococci and Contribute to Interleukin-1β Production during Infection
Source: PLoS One. 2016 Aug 10;11(8):e0160249. doi: 10.1371/journal.pone.0160249 (PMC4980021; doi:10.1371/journal.pone.0160249)
Supplement: S1 Table — (PDF) [file pone.0160249.s004.pdf]

S1 Table. Effect of anti-Ly6G treatment on blood polymorphonuclear leukocytes counts

	24 h	48 h	72 h
control IgG	6.1±0.5[a]	7.3±0.5	5.8±0.7
anti-Ly6G	0.5±0.2	0.6±0.3	0.7±0.3

[a] Percentage of $Ly6G^{+}$ cells in peripheral blood. All the values are means ± SD
