# Supplementary material for: Neutrophils Directly Recognize Group B Streptococci and Contribute to Interleukin-1β Production during Infection
Source: PLoS One. 2016 Aug 10;11(8):e0160249. doi: 10.1371/journal.pone.0160249 (PMC4980021; doi:10.1371/journal.pone.0160249)
Supplement: S2 Table — (PDF) [file pone.0160249.s005.pdf]

S2 Table. Effect of removal of macrophages with anti $CD115^{+}$-coated beads on purity of bone marrow-derived neutrophil preparations

	before	after
$F4/80^{+}$	6.7 ± 0.8[a]	0.8 ± 0.05
$Ly6G^{+}$	89 ± 3.7	95 ± 2.7
$F4/80^{-}Ly6G^{-}$	2.8 ± 0.9	1.5 ±0.7

[a] Percentage of cells. All the values are means ± SD
